# ‘Modified STOPP-START criteria for Sri Lanka’; translating to a resource limited healthcare setting by Delphi consensus

**DOI:** 10.1186/s12877-019-1293-x

**Published:** 2019-10-22

**Authors:** N. R. Samaranayake, A. Balasuriya, G. H. Fernando, D. Samaraweera, L. G. T. Shanika, J. K. P. Wanigasuriya, C. N. Wijekoon, C. A. Wanigatunge

**Affiliations:** 1 Department of Pharmacy and Pharmaceutical Sciences, Faculty of Allied Health Sciences, University of Sri Jayewardenepura, Nugegoda, Sri Lanka; 2 Hemas Hospitals, Wattala, Sri Lanka; 3 Department of Pharmacology, Faculty of Medical Sciences, University of Sri Jayewardenepura, Nugegoda, Sri Lanka; 4 Colombo South Teaching Hospital, Kalubowila, Sri Lanka; 5 Department of Medicine, Faculty of Medical Sciences, University of Sri Jayewardenepura, Nugegoda, Sri Lanka

**Keywords:** STOPP/START criteria, Delphi validation, Resource limited setting, Older adults, Sri Lanka

## Abstract

**Background:**

‘Screening tool of older people’s prescriptions (STOPP) and screening tool to alert to right treatment (START)’ criteria is a useful tool to assess the appropriateness of medicines among older adults. However, the original STOPP/START criteria developed in the West, may not be directly applicable to resource limited healthcare settings like Sri Lanka. Hence, we aimed to modify STOPP/START criteria (Version 2) to suit Sri Lanka.

**Method:**

Two investigators (a clinical pharmacologist and a pharmacist) reviewed and flagged criteria that were unfeasible to Sri Lanka based on their previous research experiences on using STOPP/START version 1. A Delphi consensus methodology was conducted among six experts, including geriatricians, clinical pharmacologists, physicians and a pharmacist, to review and assess each criterion (including the ones flagged by the researchers) for suitability to Sri Lanka.

**Results:**

Two Delphi validation rounds were conducted. A final meeting was held with the participation of all experts to resolve disagreements and to establish 100% consensus. The expert panel agreed on a list of 105 criteria, including 70 STOPP and 35 START criteria, indicating an 8% reduction in criteria compared to the original version. Modifications included complete removal (*n* = 11), re-wording (*n* = 25), splitting (*n* = 1) of original criteria and adding a new criterion (*n* = 1). Main reasons for modifications were unavailability of some medicines in the country, unavailability or inaccessibility of specific clinical information required for assessment of criteria, and adherence to treatment guidelines commonly used in the country.

**Conclusion:**

A list of ‘Modified STOPP/START criteria for Sri Lanka’ was developed. These criteria are currently being validated through a multi-centre study.

## Background

Older adults are more prone to drug related problems as most are taking a number of medicines for multiple comorbidities [1, 2]. Their physiology is different to that of a young adult and normal doses of medicine recommended for healthy adults may not be appropriate for them [1]. Some diseases which are more prone among older adults such as liver and kidney diseases may alter metabolism and excretion of medicines and necessitate dose adjustments, substitution or even addition of other medicines [1, 3]. Further, older adults may be suffering with functional problems such as physical incapacities, hearing and visual impairment and reduced state of comprehension and retention of information which would make them more prone to medication errors [4]. Thus, the category of older adults are a special group in our society who require continuous monitoring and support in terms of their medicines.

Medicines of older adults need to be managed on an individual basis as complications of one may be vastly different from another. While individualized care is a must in older adults to reduce drug related problems, screening for potential inappropriate prescribing would be of help in identifying those who need special care. Beers’ and Screening Tool of Older Persons’ Prescriptions (STOPP) and Screening Tool to Alert Doctors to Right Treatment (START) are simple, quick to apply, and objective explicit criteria used to assess appropriateness of medicines, but Beers’ criteria have not been validated outside North America [5]. STOPP/START criteria are closer to the Sri Lankan healthcare system than Beers’.

STOPP/START will help to identify those with potential problems in their prescriptions for the purpose of referring to appropriate specialist advice [6]. In fact, prioritizing older adults with drug related problems is important for optimal use of healthcare services. STOPP/START identifies potentially inappropriate prescriptions (PIPs) [4, 6, 7]. ‘Screening tool of older people’s prescriptions (STOPP)’ criteria will help to identify potentially inappropriate medicines (PIMs) and ‘Screening tool to alert to right treatment (START)’ will help to identify potential prescription omissions (PPOs). The latest version revised in 2014, consists of 114 criteria, including 80 STOPP criteria and 34 START criteria [8, 9].

Explicit criteria used to assess medication appropriateness in a country needs to suit its healthcare system. Most criteria are developed in the West and may not be completely transferable to resource limited settings such as the healthcare system in Sri Lanka. The prevalence of older adults in Sri Lanka is expected to rise above 30% of the population by the end of 2030 and is expected to contribute to the increase in the prevalence of non-communicable disease burden in Sri Lanka [10]. This increasing trend will inevitably increase the demand for healthcare services in Sri Lanka. The pool of human resources for healthcare has increased over the last 10 years, but the skill mix is imbalanced including lack of specialists. The overall healthcare cost reported in 2012 is expected to double by 2020 causing further pressure on available resources for the provision of healthcare in Sri Lanka [10]. Resource limitations result in limited access to medicines, equipment, supplies, devices, less-developed infrastructure and limited number of trained healthcare professionals [11]. Previous experiences of our research group on using STOPP/START criteria (Version 1; 2008) in a hospital and community setting in Sri Lanka, where both state and private healthcare services co-exist, revealed several issues [12]. Users of STOPP/START criteria as a tool to detect PIPs are only expected to detect potential prescription problems based on information available on medical documents, and a comprehensive patient history. We experienced problems when assessing some criteria due to unavailability of some information due to poor patient record keeping practises in Sri Lanka. Some health information were not routinely documented by prescribers, while some information were not available at the community level as medical records were retained in the hospital. Some criteria were redundant as they were related to medicines which are not registered in Sri Lanka, or is not routinely used due to the availability of cheaper options [12]. Most of the criteria that caused these practical issues hadn’t changed in STOPP/START version 2 (2014).

Given these limitations, we identified the need for modification and re-validation of the existing STOPP/START criteria for low resource settings like Sri Lanka. This paper aims to discuss the process of modifying the STOPP/START criteria for Sri Lanka through a Delphi consensus methodology.

## Method

A Delphi consensus methodology was used among a panel of six experts [13], including geriatricians (*N* = 2), clinical pharmacologists (*N* = 2), a specialist in internal medicine (*N* = 1) and a pharmacist (*N* = 1) to assess the suitability of the STOPP/START criteria (Version 2; 2014) for Sri Lanka. The expert group represented clinical pharmacologists from academia, and clinicians from tertiary healthcare hospitals (both state and private owned). The STOPP/START criteria (Version 2; 2014) was used as the working document with permission from the original authors. Two independent investigators who facilitated the Delphi process (one clinical pharmacologist and one pharmacist) had previous experience on using STOPP/START criteria (version 1;2008), among 468 community dwelling older patients in Sri Lanka [12]. Based on this previous experience, the investigators flagged criteria that are clearly irrelevant or impractical in the Sri Lankan healthcare setting with justification. They also modified the wording of some criteria to increase clarity and relevance to Sri Lanka. The original STOPP/START criteria (Version 2;2014) including the clearly marked modifications/eliminations was used as the first draft for circulation among the experts in Round 1. A five point Likert’s scale where ‘1’ indicated ‘Strongly agree’ and ‘5’ indicated ‘Strongly disagree’ was used for scoring. The experts were first invited for the study though electronic mail and the first draft was circulated among them through electronic mail once the invitation was accepted. The experts were asked to indicate their agreement/disagreement considering the validity of the criteria in principle, and its relevance to Sri Lanka. In addition to the Likert’s scale response, the experts were asked to write reasons upon disagreement to any of the criteria. Results of the first round were analysed and criteria with a median score of 1 or 2, and a 75th centile value of not > 2.5, were retained [8]. Criteria with a higher median value were rejected. The retained criteria were used to make the second draft of the STOPP/START criteria for circulation in Round 2. Results of the second round were also analysed in the same manner as in Round 1. Criteria with 100% agreement among all experts were retained. Criteria with disagreements, and criteria rejected in both first and second rounds were collated for discussion at a face to face meeting among the six experts to confirm their opinion. Discrepancies in opinions regarding each of these criteria were discussed one by one until 100% agreement was reached among all experts on criteria to either retain or reject.

## Results

The Modified STOPP/START criteria for Sri Lanka consisted of 105 criteria, including 70 STOPP criteria and 35 START criteria after two Delphi validation rounds and a final meeting held with the participation of all experts to resolve disagreements and to establish 100% consensus. Modifications included complete removal of 10 STOPP criteria and one START criterion, splitting of one START criterion in to two, adding one new criterion to the START section, and re-wording of 25 criteria (17 STOPP and 8 START). A summary of the types of modifications and main reasons for modifications of these criteria are shown in Table 1. Eight original criteria which were inconclusive in initial rounds were retained with 100% agreement at the final meeting. More details of modifications made to the STOPP/START version 2 are shown in Additional file 1: Table S1.

**Table 1:** Types of modifications to criteria and related reasons

Type of modification	Reason for modification	Criteria (Original identification)	Number of criteria modified
STOPP criteria			
Rewording by removing specific words	Medical documentation may not contain this information	B2, B13	6
	Unnecessary	B6	
	Possible difficulty in assessing as an explicit criterion	B8	
	Some medicines not registered in Sri Lanka	B10, B13, D3	
Reworded by addition of some words	To increase clarity	C10, D12, E4, G5, H1, H3	8
	Medicine use justified in special cases	D5	
	Better treatment options are not available in Sri Lanka in state sector	D14	
Reworded by changing specific words	To match tablet strength/s registered in Sri Lanka	C1	3
	To match current treatment guidelines [13]	C8, C9	
Removed	Medicine not registered in Sri Lanka	C7	10
	Better medicine options are not available in Sri Lankan in state sector	D2, D7	
	Not commonly used in Sri Lanka – Simplify STOPP/START	D11	
	Not registered in Sri Lanka	E3, K4	
	Duplication with D5	K1	
	To increase clarity	K2, L2, L3	
	Duplication with START H2	L2	
	Better suited as a START criteria	L3	
START criteria			
Reworded by changing specific words	To match tablet strength/s registered in Sri Lanka	A2	2
	To match current treatment guidelines [14, 15]	A4	
Reworded by addition of some words	To increase clarity	A7, A8	2
Reworded by removing specific words	Medical documentation may not contain this information	B2, D1	4
	Some medicines not registered in Sri Lanka	C4, E6	
Removed	Possible difficulty in assessing as an explicit criterion	C3	1
Spilt in to two criteria	To increase clarity	B1	1
Newly added	To increase clarity To avoid duplication	H group	1

Criteria were modified due to, unavailability of information in medical documentation to correctly assess the criteria (*n* = 2), presence of unnecessary words or phrases (*n* = 1), possible difficulty in assessing as an explicit criteria (*n* = 2), medicines or medicine strengths not registered in Sri Lanka (*n* = 6), less clarity (*n* = 5), medicines use justified in special cases (*n* = 1), unavailability of better treatment options in state healthcare sector in Sri Lanka (*n* = 2), matching current treatment guidelines (*n* = 3) [14–17], infrequent use in Sri Lanka (*n* = 1), duplication with other criteria (*n* = 3), and better suitability as a START criteria (*n* = 1) (Table 1).

Nine criteria were flagged by the investigators for complete removal before the Delphi Round 1, of which only two were rejected after the Delphi validation process. Four criteria were rejected in Round 1 and were endorsed as rejected by experts at the final meeting. Five other criteria were rejected at the final meeting after iterative discussions.

## Discussion

STOPP/START criteria version 2 was modified to suit the needs of a healthcare system operated in a resource limited setting such as Sri Lanka. The modified criteria consisted of 105 criteria including 70 STOPP and 35 START criteria, indicating a 12.5% reduction in STOPP criteria and a 3% increase in START criteria compared to the original version. Among many reasons for modification, removal of medicines or medicine strengths not registered in Sri Lanka, and re-wording of criteria to improve clarity were the most common.

STOPP/START criteria are useful to screen potential medicine related problems among older adults attending healthcare institutions of any level. It is especially useful in resource limited healthcare settings like Sri Lanka [10, 11, 18] where the healthcare system is different in many ways to resource rich countries. Sri Lankan citizens have many treatment options including allopathic, and non-allopathic options such as Ayurveda, Siddha and Unani. Allopathic health services are offered by both, state institutions, free of charge, and by private institutions for a fee. State owned allopathic health facilities range from primary to tertiary care level but operate based primarily on an essential medicines list due to limited availability of resources. As there are a variety of health service available, Sri Lankans are often found to use more than one option and also seek treatment from multiple physicians at any given time, leading to problems such as medicine duplications and medicine interactions. Statistics indicate that most Sri Lankans use private sector health services for outpatient care, and state sector health services for inpatient care [10, 11, 18]. However, there is no formal method of linking health records of the state and private sectors unless the patient is knowledgeable enough to coordinate handwritten documents provided by each provider. Most state sector health records are stored at the hospitals and patients do not have a complete medical document in hand. A complicated system of this nature could give rise to many medication related problems simply through poor coordination among healthcare providers. In Sri Lanka, patients also have direct access to any level of healthcare, and do not have to go through a sequential screening from primary to tertiary levels. Due to this reason, tertiary care level health services are unnecessarily over-burdened, while primary healthcare services are grossly underutilized, and less well developed. The large patient numbers anticipating tertiary care health services may not receive due attention owing to limited resources both in terms of availability of medicines and healthcare professionals. This limitation too could greatly aggravate the use of inappropriate medicines or omission of required medicines among patients, the aged being more vulnerable. Thus, screening for potentially inappropriate prescriptions by healthcare professionals first using the modified STOPP/START criteria, and then referral of selected patients for individualized care by specialists, would be an effective way forward in ensuring patient safety in a setting like Sri Lanka. However, given the limitations on access to complete medical records of patients, and poor documentation practices, the tool used for the assessment of inappropriate medicines should be simple but comprehensive.

There are some limitations in this study that must be acknowledged. The STOPP/START criteria were modified based on characteristics of the healthcare system of Sri Lanka and hence may not be directly applicable to other resource limited healthcare settings. If the modified tool is to be used in similar resource limited countries, it should be validated in the respective countries in a similar manner. The expert panel was limited to only six experts which is a small group but evidence supports that this is an acceptable number for a scientifically valid Delphi process [13]. Although all experts were involved with care of older adults, there was limited representation from primary healthcare services. National treatment guidelines are only available for some diseases in Sri Lanka and this was also a limitation when validating the criteria.

## Conclusion

In conclusion, the STOPP/START criteria were modified to overcome common issues encountered when applying the tool in low resource healthcare settings like Sri Lanka. The modified criteria included 105 criteria; 70 STOPP and 35 START criteria, indicating an 8% reduction in criteria compared to the original version. The simplified and more relevant set of criteria is expected to yield a more realistic outcome when assessing for inappropriate medication among older adults in Sri Lanka. It is expected that the modified tool will be used by medical officers or pharmacists to screen out older patients with potentially inappropriate medicines to refer for specialist care. Once validated, we hope to make the Modified STOPP/START criteria for Sri Lanka available online and accessible without a fee for healthcare professionals in Sri Lanka. We also hope to introduce this tool at undergraduate level in a relevant section of the Bachelor of Pharmacy curriculum. The modified tool could also be largely applicable to other countries with similar healthcare systems but should always be validated to the respective country before application in large scale.

## Supplementary information

**Additional file 1: Table S1.** Details of modifications in the ‘Modified STOPP/START criteria’ for Sri Lanka.

## Data Availability

The datasets used and/or analyzed during the current study are available from the corresponding author on reasonable request.

## Abbreviations

PIMs: Potentially inappropriate medicine
PIPs: Potentially inappropriate prescriptions
PPOs: Potential prescription omissions
START: Screening tool to alert to right treatment
STOPP: Screening tool of older people’s prescriptions
