# Supplementary material for: ‘Modified STOPP-START criteria for Sri Lanka’; translating to a resource limited healthcare setting by Delphi consensus
Source: BMC Geriatr. 2019 Oct 22;19:282. doi: 10.1186/s12877-019-1293-x (PMC6805460; doi:10.1186/s12877-019-1293-x)
Supplement: Supplementary file 1 — Additional file 1: Table S1. Details of modifications in the ‘Modified STOPP/START criteria’ for Sri Lanka. [file 12877_2019_1293_MOESM1_ESM.docx]

**Table S1: Details of modifications in the ‘Modified STOPP/START criteria’ for Sri Lanka**

| **Serial No** | **Original identifier** | **STOPP/START original version (Version 2)**^7^ | **‘Modified STOPP/START criteria for Sri Lanka** | | **Outcome of Delphi** | **Reason if any** |
| --- | --- | --- | --- | --- | --- | --- |
|  |  | **STOPP Criteria** | | |  |  |
|  |  | **Section A: Indication of medication** | | |  |  |
|  | A1 | Any drug prescribed without an evidence-based clinical indication | Any drug prescribed without an evidence-based clinical indication | | Not changed | - |
|  | A2 | Any drug prescribed beyond the recommended duration, where treatment duration is well defined | Any drug prescribed beyond the recommended duration, where treatment duration is well defined. | | Not changed | - |
|  | A3 | Any duplicate drug class prescription e.g. two concurrent NSAIDs, SSRIs, loop diuretics, ACE inhibitors, anticoagulants | Any duplicate drug class prescription e.g. two concurrent NSAIDs, SSRIs, loop diuretics, ACE inhibitors, anticoagulants | | Not changed | - |
|  |  | **Section B: Cardiovascular System** | | |  |  |
|  | B1 | Digoxin for heart failure with normal systolic ventricular function | Digoxin for heart failure with normal systolic ventricular function | | Not changed | - |
|  | B2 | Verapamil or diltiazem with NYHA Class III or IV heart failure | Verapamil or diltiazem in those with heart failure | | Reworded | Medical documentation may not contain information regarding the stage of heart failure |
|  | B3 | Beta-blocker in combination with verapamil or diltiazem | Beta-blocker in combination with verapamil or diltiazem | | Not changed | - |
|  | B4 | Beta blocker with bradycardia (< 50/min), type II heart block or complete heart block | Beta blocker with bradycardia (< 50/min), type II heart block or complete heart block | | Not changed | - |
|  | B5 | Amiodarone as first-line antiarrhythmic therapy in supraventricular tachyarrhythmias | Amiodarone as first-line antiarrhythmic therapy in supraventricular tachyarrhythmias | | Not changed | - |
|  | B6 | Loop diuretic as first-line treatment for hypertension | Loop diuretic as treatment for hypertension | | Reworded | The word ‘first line’ unnecessary |
|  | B7 | Loop diuretic for dependent ankle oedema without clinical, biochemical evidence or radiological evidence of heart failure, liver failure, nephrotic syndrome or renal failure | Loop diuretic for dependent ankle oedema without clinical, biochemical evidence or radiological evidence of heart failure, liver failure, nephrotic syndrome or renal failure | | Not changed |  |
|  | B8 | Thiazide diuretic with current significant hypokalaemia (i.e. serum K+ < 3.0 mmol/l), hyponatraemia (i.e. serum Na+ < 130 mmol/l) hypercalcaemia (i.e. corrected serum calcium > 2.65 mmol/l) or with a history of gout | Thiazide diuretic with current significant hypokalaemia (i.e. serum K+ < 3.0 mmol/l), hyponatraemia (i.e. serum Na+ < 130 mmol/l) hypercalcaemia (i.e. corrected serum calcium > 2.65 mmol/l) | | Reworded | 'History of gout’ difficult to assess as an explicit criteria |
|  | B9 | Loop diuretic for treatment of hypertension with concurrent urinary incontinence | Loop diuretic for treatment of hypertension with concurrent urinary incontinence | | Not changed | - |
|  | B10 | Centrally-acting antihypertensives (e.g. methyldopa, clonidine, moxonidine, rilmenidine, guanfacine), unless clear intolerance of, or lack of efficacy with, other classes of antihypertensives | Centrally-acting antihypertensives (e.g. methyldopa, clonidine), unless clear intolerance of, or lack of efficacy with, other classes of antihypertensives | | Reworded | ‘Moxonidine, rilmenidine, and guanfacine’ not registered in  Sri Lanka |
|  | B11 | ACE inhibitors or Angiotensin Receptor Blockers in patients with hyperkalaemia | ACE inhibitors or Angiotensin Receptor Blockers in patients with hyperkalaemia. | | Not changed | - |
|  | B12 | Aldosterone antagonists (e.g. spironolactone, eplerenone) with concurrent potassium-conserving drugs (e.g. ACEI’s, ARB’s, amiloride, triamterene) without monitoring of serum potassium | Aldosterone antagonists (e.g. spironolactone, eplerenone) with concurrent potassium-conserving drugs (e.g. ACEI’s, ARB’s, amiloride, triamterene) without monitoring of serum potassium | | Not changed | - |
|  | B13 | Phosphodiesterase type-5 inhibitors (e.g. sildenafil, tadalafil, vardenafil) in severe heart failure characterized by hypotension i.e. systolic BP < 90 mmHg, or concurrent nitrate therapy for angina (risk of cardiovascular collapse) | Phosphodiesterase type-5 inhibitors (e.g. sildenafil) with concurrent nitrate therapy for angina | | Reworded | Tadalafil, vardenafil are not registered in Sri Lanka  Medical documentation may not contain the severity of heart failure |
|  |  | **Section C: Antiplatelet/Anticoagulant Drugs** | | |  |  |
|  | C1 | Long-term aspirin at doses greater than 160 mg per day | Long-term aspirin at doses greater than 150 mg per day | | Reworded | Tablet strength registered in Sri Lanka is 150mg and 300mg |
|  | C2 | Aspirin with a past history of peptic ulcer disease without concomitant PPI | Aspirin with a past history of peptic ulcer disease without concomitant PPI | | Not changed | - |
|  | C3 | Aspirin, clopidogrel, dipyridamole, vitamin K antagonists, direct thrombin inhibitors or factor Xa inhibitors with concurrent significant bleeding risk, i.e. uncontrolled severe hypertension, bleeding diathesis, recent non-trivial spontaneous bleeding | Aspirin, clopidogrel, dipyridamole, vitamin K antagonists, direct thrombin inhibitors or factor Xa inhibitors with concurrent significant bleeding risk, i.e. uncontrolled severe hypertension, bleeding diathesis, recent non-trivial spontaneous bleeding | | Not changed | - |
|  | C4 | Aspirin plus clopidogrel as secondary stroke prevention, unless the patient has a coronary stent(s) inserted in the previous 12 months or concurrent acute coronary syndrome or has a high grade symptomatic carotid arterial stenosis | Aspirin plus clopidogrel as secondary stroke prevention, unless the patient has a coronary stent(s) inserted in the previous 12 months or concurrent acute coronary syndrome or has a high grade symptomatic carotid arterial stenosis | | Not changed | - |
|  | C5 | Aspirin in combination with vitamin K antagonist, direct thrombin inhibitor or factor Xa inhibitors in patients with chronic atrial fibrillation | Aspirin in combination with vitamin K antagonist, direct thrombin inhibitor or factor Xa inhibitors in patients with chronic atrial fibrillation | | Not changed | - |
|  | C6 | Antiplatelet agents with vitamin K antagonist, direct thrombin inhibitor or factor Xa inhibitors in patients with stable coronary, cerebrovascular or peripheral arterial disease | Antiplatelet agents with vitamin K antagonist, direct thrombin inhibitor or factor Xa inhibitors in patients with stable coronary, cerebrovascular or peripheral arterial disease | | Not changed | - |
|  | C7 | Ticlopidine in any circumstances (clopidogrel and prasugrel have similar efficacy, stronger evidence and fewer side-effects). | ~~-~~ | | Removed | Medicine not registered in Sri Lanka |
|  | C8 | Vitamin K antagonist, direct thrombin inhibitor or factor Xa inhibitors for first deep venous thrombosis without continuing provoking risk factors (e.g. thrombophilia) for > 6 months | Vitamin K antagonist, direct thrombin inhibitor or factor Xa inhibitors for first deep venous thrombosis without continuing provoking risk factors for > 3 months | | Reworded | To match current treatment guidelines |
|  | C9 | Vitamin K antagonist, direct thrombin inhibitor or factor Xa inhibitors for first pulmonary embolus without continuing provoking risk factors (e.g. thrombophilia) for > 12 months | Vitamin K antagonist, direct thrombin inhibitor or factor Xa inhibitors for first pulmonary embolus without continuing provoking risk factors for >3 months | | Reworded | To match current treatment guidelines |
|  | C10 | NSAID and vitamin K antagonist, direct thrombin inhibitor or factor Xa inhibitors in combination | NSAID (excluding aspirin 75mg for an appropriate indication) and vitamin K antagonist, direct thrombin inhibitor or factor Xa inhibitors in combination | | Reworded | Avoid confusion |
|  | C11 | NSAID with concurrent antiplatelet agent(s) without PPI prophylaxis | NSAID with concurrent antiplatelet agent(s) without PPI prophylaxis | | Not changed | - |
|  |  | **Section D: Central Nervous System and Psychotropic Drugs** | | |  |  |
|  | D1 | TriCyclic Antidepressants (TCAs) with dementia, narrow angle glaucoma, cardiac conduction abnormalities, prostatism, or prior history of urinary retention | Tricyclic antidepressants (TCAs) with dementia, narrow angle glaucoma, cardiac conduction abnormalities, prostatism, or prior history of urinary retention | | Not changed | - |
|  | D2 | Initiation of TriCyclic Antidepressants (TCAs) as first-line antidepressant treatment |  | | Removed | Better medicine options are not available in Sri Lankan state hospitals |
|  | D3 | Neuroleptics with moderate-marked antimuscarinic/anticholinergic effects (chlorpromazine, clozapine, flupenthixol, fluphenzine, pipothiazine, promazine, zuclopenthixol) with a history of prostatism or previous urinary retention | Neuroleptics with moderate-marked antimuscarinic/anticholinergic effects (chlorpromazine, clozapine, flupenthixol, zuclopenthixol) with a history of prostatism or previous urinary retention | | Reworded | Fluphenzine, pipothiazine, and promazine are not registered in Sri Lanka |
|  | D4 | Selective serotonin re-uptake inhibitors (SSRI’s) with current or recent significant hyponatraemia i.e. serum Na+ < 130 mmol/l | Selective serotonin re-uptake inhibitors (SSRI’s) with current or recent significant hyponatraemia i.e. serum Na+ < 130 mmol/l | | Not changed | - |
|  | D5 | Benzodiazepines for ≥ 4 weeks | Benzodiazepines for ≥ 4 weeks unless prescribed for treatment of epilepsy | | Reworded | May be used for epilepsy under special conditions |
|  | D6 | Antipsychotics (i.e. other than quetiapine or clozapine) in those with parkinsonism or Lewy Body Disease | Antipsychotics (i.e. other than quetiapine or clozapine) in those with parkinsonism or Lewy Body Disease | | Not changed | - |
|  | D7 | Anticholinergics/antimuscarinics to treat extra-pyramidal side-effects of neuroleptic medications |  | | Removed | Better medicine options are not available in Sri Lankan state hospitals |
|  | D8 | Anticholinergics/antimuscarinics in patients with delirium or dementia | Anticholinergics/antimuscarinics in patients with delirium or dementia | | Not changed | - |
|  | D9 | Neuroleptic antipsychotic in patients with behavioural and psychological symptoms of dementia (BPSD) unless symptoms are severe and other non-pharmacological treatments have failed | Neuroleptic antipsychotic in patients with behavioural and psychological symptoms of dementia (BPSD) unless symptoms are severe and other non-pharmacological treatments have failed | | Not changed | - |
|  | D10 | Neuroleptics as hypnotics, unless sleep disorder is due to psychosis or dementia | Neuroleptics as hypnotics, unless sleep disorder is due to psychosis or dementia | | Not changed | - |
|  | D11 | Acetylcholinesterase inhibitors with a known history of persistent bradycardia (< 60 beats/min.), heart block or recurrent unexplained syncope or concurrent treatment with drugs that reduce heart rate such as beta-blockers, digoxin, diltiazem, verapamil |  | | Removed | Not commonly used in Sri Lanka  Removed to simplify criteria |
|  | D12 | Phenothiazines as first-line treatment, since safer and more efficacious alternatives exist | Phenothiazines as first-line treatment for psychotic disorders, since safer and more efficacious alternatives exist | | Reworded | Avoid confusion |
|  | D13 | Levodopa or dopamine agonists for benign essential tremor | Levodopa or dopamine agonists for benign essential tremor | | Not changed | - |
|  | D14 | First-generation antihistamines | First-generation antihistamines if newer antihistamines are available | | Reworded | Newer antihistamines may not be available at times in Sri Lankan state hospitals |
|  |  | **Section E: Renal System** | | |  |  |
|  | E1 | Digoxin at a long-term dose greater than 125µg/day if eGFR < 30 ml/min/1.73m^2^ | Digoxin at a long-term dose greater than 125µg/day if eGFR < 30 ml/min/1.73m^2^ | | Not changed | - |
|  | E2 | Direct thrombin inhibitors (e.g. dabigatran) if eGFR < 30 ml/min/1.73m^2^ | Direct thrombin inhibitors (e.g. dabigatran) if eGFR < 30 ml/min/1.73m^2^ | | Not changed | - |
|  | E3 | Factor Xa inhibitors (e.g. rivaroxaban, apixaban) if eGFR < 15 ml/min/1.73m2 |  | | Removed | Medicine not registered in Sri Lanka |
|  | E4 | NSAID’s if eGFR < 50 ml/min/1.73m2 | NSAIDs (excluding aspirin 75 mg) if eGFR < 50 ml/min/1.73m2 | | Reworded | Increase clarity |
|  | E5 | Colchicine if eGFR < 10 ml/min/1.73m2 | Colchicine if eGFR < 10 ml/min/1.73m2 | | Not changed | - |
|  | E6 | Metformin if eGFR < 30 ml/min/1.73m2 | Metformin if eGFR < 30 ml/min/1.73m2 | | Not changed | - |
|  |  | **Section F:** **Gastrointestinal System** | | |  |  |
|  | F1 | Prochlorperazine or metoclopramide with Parkinsonism | Prochlorperazine or metoclopramide with Parkinsonism | | Not changed | - |
|  | F2 | PPI for uncomplicated peptic ulcer disease or erosive peptic oesophagitis at full therapeutic dosage for > 8 weeks | PPI for uncomplicated peptic ulcer disease or erosive peptic oesophagitis at full therapeutic dosage for > 8 weeks | | Not changed | - |
|  | F3 | Drugs likely to cause constipation (e.g. antimuscarinic/anticholinergic drugs, oral iron, opioids, verapamil, aluminium antacids) in patients with chronic constipation where non-constipating alternatives are available | Drugs likely to cause constipation (e.g. antimuscarinic/anticholinergic drugs, oral iron, opioids, verapamil, aluminium antacids) in patients with chronic constipation where non-constipating alternatives are available | | Not changed | - |
|  | F4 | Oral elemental iron doses greater than 200 mg daily (e.g. ferrous fumarate> 600 mg/day, ferrous sulphate > 600 mg/day, ferrous gluconate> 1800 mg/day). | Oral elemental iron doses greater than 200 mg daily (e.g. ferrous fumarate> 600 mg/day, ferrous sulphate > 600 mg/day, ferrous gluconate> 1800 mg/day). | | Not changed | - |
|  |  | **Section G**: **Respiratory System** | | |  |  |
|  | G1 | Theophylline as monotherapy for COPD | Theophylline as monotherapy for COPD | | Not changed | - |
|  | G2 | Systemic corticosteroids instead of inhaled corticosteroids for maintenance therapy in moderate-severe COPD | Systemic corticosteroids instead of inhaled corticosteroids for maintenance therapy in moderate-severe COPD | | Not changed | - |
|  | G3 | Anti-muscarinic bronchodilators (e.g. ipratropium, tiotropium) with a history of narrow angle glaucoma or bladder outflow obstruction | Anti-muscarinic bronchodilators (e.g. ipratropium, tiotropium) with a history of narrow angle glaucoma or bladder outflow obstruction | | Not changed | - |
|  | G4 | Benzodiazepines with acute or chronic respiratory failure i.e. pO2 < 8.0 kPa ± pCO2 > 6.5 kPa | Benzodiazepines with symptomatic obstructive lung disease | | Reworded | Increase clarity by simplification |
|  |  | **Section H: Musculoskeletal System** | | |  |  |
|  | H1 | Non-steroidal anti-inflammatory drug (NSAID) other than COX-2 selective agents with history of peptic ulcer disease or gastrointestinal bleeding, unless with concurrent PPI or H2 antagonist (risk of peptic ulcer relapse). | In those with history of peptic ulcer disease or gastrointestinal bleeding, non-steroidal anti-inflammatory drug (NSAID) (including aspirin in any dose) and COX-2 selective agents unless with concurrent PPI or H2 antagonist | | Reworded | Increase clarity |
|  | H2 | NSAID with severe hypertension or severe heart failure | NSAID (excluding aspirin 75 mg) with severe hypertension or severe heart failure | | Reworded | Increase clarity |
|  | H3 | Long-term use of NSAID (>3 months) for symptom relief of osteoarthritis pain where paracetamol has not been tried | Long-term use of NSAID (>3 months) for symptom relief of osteoarthritis pain where paracetamol has not been tried | | Not changed | - |
|  | H4 | Long-term corticosteroids (>3 months) as monotherapy for rheumatoid arthritis | Long-term corticosteroids (>3 months) as monotherapy for rheumatoid arthritis | | Not changed | - |
|  | H5 | Corticosteroids (other than periodic intra-articular injections for mono-articular pain) for osteoarthritis | Corticosteroids (other than periodic intra-articular injections for mono-articular pain) for osteoarthritis | | Not changed | - |
|  | H6 | Long-term NSAID or colchicine (>3 months) for chronic treatment of gout where there is no contraindication to a xanthine-oxidase inhibitor (e.g. allopurinol, febuxostat) | Long-term NSAID or colchicine (>3 months) for chronic treatment of gout where there is no contraindication to a xanthine-oxidase inhibitor (e.g. allopurinol, febuxostat) | | Not changed | - |
|  | H7 | COX-2 selective NSAIDs with concurrent cardiovascular disease | COX-2 selective NSAIDs with concurrent cardiovascular disease | | Not changed | - |
|  | H8 | NSAID with concurrent corticosteroids without PPI prophylaxis | NSAID with concurrent corticosteroids without PPI prophylaxis | | Not changed | - |
|  | H9 | Oral bisphosphonates in patients with a current or recent history of upper gastrointestinal disease i.e. dysphagia, oesophagitis, gastritis, duodenitis, or peptic ulcer disease, or upper gastrointestinal bleeding | Oral bisphosphonates in patients with a current or recent history of upper gastrointestinal disease i.e. dysphagia, oesophagitis, gastritis, duodenitis, or peptic ulcer disease, or upper gastrointestinal bleeding | | Not changed | - |
|  |  | **Section I: Urogenital System** | | |  |  |
|  | I1 | 1. Antimuscarinic drugs with dementia, or chronic cognitive impairment or narrow-angle glaucoma, or chronic prostatism | Antimuscarinic drugs with dementia, or chronic cognitive impairment or narrow-angle glaucoma, or chronic prostatism | | Not changed | - |
|  | I2 | Selective alpha-1 selective alpha blockers in those with symptomatic orthostatic hypotension or micturition syncope | Selective alpha-1 selective alpha blockers in those with symptomatic orthostatic hypotension or micturition syncope | | Not changed | - |
|  |  | **System J**: **Endocrine System** | | |  |  |
|  | J1 | Sulphonylureas with a long duration of action (e.g. glibenclamide, chlorpropamide, glimepiride) with type 2 diabetes mellitus | Sulphonylureas with a long duration of action (e.g. glibenclamide, chlorpropamide, glimepiride) with type 2 diabetes mellitus. | | Not changed | - |
|  | J2 | Thiazolidenediones (e.g. rosiglitazone, pioglitazone) in patients with heart failure | Thiazolidenediones (e.g. rosiglitazone, pioglitazone) in patients with heart failure | | Not changed | - |
|  | J3 | Beta-blockers in diabetes mellitus with frequent hypoglycaemic episodes | Beta-blockers in diabetes mellitus with frequent hypoglycaemic episodes | | Not changed | - |
|  | J4 | Oestrogens with a history of breast cancer or venous thromboembolism | Oestrogens with a history of breast cancer or venous thromboembolism. | | Not changed | - |
|  | J5 | Oral oestrogens without progestogen in patients with intact uterus | Oral oestrogens without progestogen in patients with intact uterus | | Not changed | - |
|  | J6 | Androgens (male sex hormones) in the absence of primary or secondary hypogonadism | Androgens (male sex hormones) in the absence of primary or secondary hypogonadism | | Not changed | - |
|  |  | **Section K:** **Drugs that predictably increase the risk of falls in older people** | | |  |  |
|  | K1 | Benzodiazepines (sedative, may cause reduced sensorium, impair balance). | ~~-~~ | | Removed | Duplication with D5 |
|  | K2 | Neuroleptic drugs (may cause gait dyspraxia, Parkinsonism). | ~~-~~ | | Removed | Increase clarity |
|  | K3 | Vasodilator drugs (e.g. alpha-1 receptor blockers, calcium channel blockers, long-acting nitrates, ACE inhibitors, angiotensin I receptor blockers) with persistent postural hypotension i.e. recurrent drop in systolic blood pressure ≥ 20mmHg | Vasodilator drugs (e.g. alpha-1 receptor blockers, calcium channel blockers, long-acting nitrates, ACE inhibitors, angiotensin I receptor blockers) with persistent postural hypotension i.e. recurrent drop in systolic blood pressure ≥ 20mmHg | | Not changed | - |
|  | K4 | Hypnotic Z-drugs e.g. zopiclone, zolpidem, zaleplon |  | | Removed | Medicine not registered in Sri Lanka |
|  |  | **Section L: Analgesic Drugs** | | |  |  |
|  | L1 | Use of oral or transdermal strong opioids (morphine, oxycodone, fentanyl, buprenorphine, diamorphine, methadone, tramadol, pethidine, pentazocine) as first line therapy for mild pain | Use of oral or transdermal strong opioids (morphine, oxycodone, fentanyl, buprenorphine, diamorphine, methadone, tramadol, pethidine, pentazocine) as first line therapy for mild pain | | Not changed | - |
|  | L2 | Use of regular (as distinct from PRN) opioids without concomitant laxative |  | | Removed | Increase clarity and duplication with START H2 |
|  | L3 | Long-acting opioids without short-acting opioids for break-through pain |  | | Relocated to START | Better suited as a START criteria |
|  |  | **System N: Antimuscarinic/Anticholinergic Drug Burden** | | |  |  |
|  | N1 | Concomitant use of two or more drugs with antimuscarinic/anticholinergic properties (e.g. bladder antispasmodics, intestinal antispasmodics, tricyclic antidepressants, first generation antihistamines) | Concomitant use of two or more drugs with antimuscarinic/anticholinergic properties (e.g. bladder antispasmodics, intestinal antispasmodics, tricyclic antidepressants, first generation antihistamines) | | Not changed | - |
|  |  |  |  |  |  |  |

|  |  | **START Criteria** | |  |  |
| --- | --- | --- | --- | --- | --- |
|  |  | **Section A: Cardiovascular System** | |  |  |
| 1. | A1 | Vitamin K antagonists or direct thrombin inhibitors or factor Xa inhibitors in the presence of chronic atrial fibrillation. | Vitamin K antagonists or direct thrombin inhibitors or factor Xa inhibitors in the presence of chronic atrial fibrillation. | Not changed | - |
| 2. | A2 | Aspirin (75 mg – 160 mg once daily) in the presence of chronic atrial fibrillation, where Vitamin K antagonists or direct thrombin inhibitors or factor Xa inhibitors are contraindicated. | Aspirin (75mg – 150mg once daily) in the presence of chronic atrial fibrillation, where Vitamin K antagonists or direct thrombin inhibitors or factor Xa inhibitors are contraindicated. | Reworded | To match tablet strength/s registered in Sri Lanka |
| 3. | A3 | Antiplatelet therapy (aspirin or clopidogrel or prasugrel or ticagrelor) with a documented history of coronary, cerebral or peripheral vascular disease | Antiplatelet therapy (aspirin or clopidogrel or prasugrel or ticagrelor) with a documented history of coronary, cerebral or peripheral vascular disease | Not changed | - |
| 4. | A4 | Antihypertensive therapy where systolic blood pressure consistently > 160 mmHg and/or diastolic blood pressure consistently >90 mmHg; if systolic blood pressure > 140 mmHg and /or diastolic blood pressure > 90 mmHg, if diabetic. | Antihypertensive therapy where systolic blood pressure consistently > 160 mmHg and/or diastolic blood pressure consistently >=100 mmHg; if systolic blood pressure > 140 mmHg and /or diastolic blood pressure > 90 mmHg, if diabetic. | Reworded | To match current treatment guidelines |
| 5. | A5 | Statin therapy with a documented history of coronary, cerebral or peripheral vascular disease, unless the patient’s status is end-of-life or age is > 85 years | Statin therapy with a documented history of coronary, cerebral or peripheral vascular disease, unless the patient’s status is end-of-life or age is > 85 years | Not changed | - |
| 6. | A6 | Angiotensin Converting Enzyme (ACE) inhibitor with systolic heart failure and/or documented coronary artery disease. | Angiotensin Converting Enzyme (ACE) inhibitor with systolic heart failure and/or documented coronary artery disease. | Not changed | - |
| 7. | A7 | Beta-blocker with ischaemic heart disease | Beta-blocker with ischaemic heart disease in the absence of contraindications. | Reworded | To increase clarity |
| 8. | A8 | Appropriate beta-blocker (bisoprolol, nebivolol, metoprolol or carvedilol) with stable systolic heart failure | Appropriate beta-blocker (bisoprolol, nebivolol, metoprolol or carvedilol) with stable systolic heart failure in the absence of contraindications | Reworded | To increase clarity |
|  |  | **Section B: Respiratory System** | |  |  |
| 9. | B1 | Regular inhaled β2 agonist or antimuscarinic bronchodilator (e.g. ipratropium, tiotropium) for mild to moderate asthma or COPD. | Short acting inhaled beta 2 agonists to be taken as required for asthma | Reworded | Divided to two criteria and re-worded to avoid confusion |
|  |  |  | Short acting inhaled beta 2 agonists or antimuscarinic bronchodilator to be taken as required for COPD | Newly added |  |
| 10. | B2 | Regular inhaled corticosteroid for moderate-severe asthma or COPD, where FEV1 <50% of predicted value and repeated exacerbations requiring treatment with oral corticosteroids. | Regular inhaled corticosteroid for persistent asthma or COPD | Reworded | Medical documentation may not contain this information |
| 11. | B3 | Home continuous oxygen with documented chronic hypoxaemia (i.e. pO2 < 8.0 kPa or 60 mmHg or SaO2 < 89%) | Home continuous oxygen with documented chronic hypoxaemia (i.e. pO2 < 8.0 kPa or 60 mmHg or SaO2 < 89%) | Not changed | - |
|  |  | **Section C: Central Nervous System& Eyes** | |  |  |
| 12. | C1 | L-DOPA or a dopamine agonist in idiopathic Parkinson’s disease with functional impairment and resultant disability | L-DOPA or a dopamine agonist in idiopathic Parkinson’s disease with functional impairment and resultant disability | Not changed | - |
| 13. | C2 | Non-TCA antidepressant drug in the presence of persistent major depressive symptoms | Non-TCA antidepressant drug in the presence of persistent major depressive symptoms | Not changed | - |
| 14. | C3 | Acetylcholinesterase inhibitor (e.g. donepezil, rivastigmine, galantamine) for mild-moderate Alzheimer’s dementia or Lewy Body dementia (rivastigmine). |  | Removed | Possible difficulty in assessing as an explicit criteria |
| 15. | C4 | Topical prostaglandin, prostamide or beta-blocker for primary open-angle glaucoma. | Topical prostaglandin, or beta-blocker for primary open-angle glaucoma. | Reworded | Some medicines not registered in Sri Lanka |
| 16. | C5 | Selective serotonin reuptake inhibitor (or SNRI or pregabalin if SSRI contraindicated) for persistent severe anxiety that interferes with independent functioning | Selective serotonin reuptake inhibitor (or SNRI or pregabalin if SSRI contraindicated) for persistent severe anxiety that interferes with independent functioning | Not changed | - |
| 17. | C6 | Dopamine agonist (ropinirole or pramipexole or rotigotine) for Restless Legs Syndrome, once iron deficiency and severe renal failure have been excluded | Dopamine agonist (ropinirole or pramipexole or rotigotine) for Restless Legs Syndrome, once iron deficiency and severe renal failure have been excluded | Not changed | - |
|  |  | **Section D: Gastrointestinal System** | |  |  |
| 18. | D1 | Proton Pump Inhibitor with severe gastro-oesophageal reflux disease or peptic stricture requiring dilatation. | Proton Pump Inhibitor with severe gastro-oesophageal reflux disease | Reworded | Medical documentation may not contain this information |
| 19. | D2 | Fibre supplements (e.g. bran, ispaghula, methylcellulose, sterculia) for diverticulosis with a history of constipation | Fibre supplements (e.g. bran, ispaghula, methylcellulose, sterculia) for diverticulosis with a history of constipation | Not changed | - |
|  |  | **Section E: Musculoskeletal System** | |  |  |
| 20. | E1 | Disease-modifying anti-rheumatic drug (DMARD) with active, disabling rheumatoid disease | Disease-modifying anti-rheumatic drug (DMARD) with active, disabling rheumatoid disease. | Not changed | - |
| 21. | E2 | Bisphosphonates and vitamin D and calcium in patients taking long-term systemic corticosteroid therapy | Bisphosphonates and vitamin D and calcium in patients taking long-term systemic corticosteroid therapy | Not changed | - |
| 22. | E3 | Vitamin D and calcium supplement in patients with known osteoporosis and/or previous fragility fracture(s) and/or (Bone Mineral Density T-scores more than -2.5 in multiple site | Vitamin D and calcium supplement in patients with known osteoporosis and/or previous fragility fracture(s) and/or (Bone Mineral Density T-scores more than -2.5 in multiple sites) | Not changed | - |
| 23. | E4 | Bone anti-resorptive or anabolic therapy (e.g. bisphosphonate, strontium ranelate, teriparatide, denosumab) in patients with documented osteoporosis, where no pharmacological or clinical status contraindication exists (Bone Mineral Density T-scores -> 2.5 in multiple sites) and/or previous history of fragility fracture(s) | Bone anti-resorptive or anabolic therapy (e.g. bisphosphonate, strontium ranelate, teriparatide) in patients with documented osteoporosis, where no pharmacological or clinical status contraindication exists (Bone Mineral Density T-scores -> 2.5 in multiple sites) and/or previous history of fragility fracture(s) | Not changed | - |
| 24. | E5 | Vitamin D supplement in older people who are housebound or experiencing falls or with osteopenia (Bone Mineral Density T-score is > -1.0 but < -2.5 in multiple sites) | Vitamin D supplement in older people who are housebound or experiencing falls or with osteopenia (Bone Mineral Density T-score is > -1.0 but < -2.5 in multiple sites) | Not changed | - |
| 25. | E6 | Xanthine-oxidase inhibitors (e.g. allopurinol, febuxostat) with a history of recurrent episodes of gout | Xanthine-oxidase inhibitors (e.g. allopurinol) with a history of recurrent episodes of gout | Reworded | Some medicines not registered in Sri Lanka |
| 26. | E7 | Folic acid supplement in patients taking methotexate | Folic acid supplement in patients taking methotrexate | Not changed | - |
|  |  | **Section F: Endocrine System** | |  |  |
| 27. | F1 | ACE inhibitor or Angiotensin Receptor Blocker (if intolerant of ACE inhibitor) in diabetes with evidence of renal disease i.e. dipstick proteinuria or microalbuminuria (>30mg/24 hours) with or without serum biochemical renal impairment | ACE inhibitor or Angiotensin Receptor Blocker (if intolerant of ACE inhibitor) in diabetes with evidence of renal disease i.e. dipstick proteinuria or microalbuminuria (>30mg/24 hours) with or without serum biochemical renal impairment | Not changed | - |
|  |  | **Section G: Urogenital System** | |  |  |
| 28. | G1 | Alpha-1 receptor blocker with symptomatic prostatism, where prostatectomy is not considered necessary | Alpha-1 receptor blocker with symptomatic prostatism, where prostatectomy is not considered necessary | Not changed | - |
| 29. | G2 | 5-alpha reductase inhibitor with symptomatic prostatism, where prostatectomy is not considered necessary. | 5-alpha reductase inhibitor with symptomatic prostatism, where prostatectomy is not considered necessary. | Not changed | - |
| 30. | G3 | Topical vaginal oestrogen or vaginal oestrogen pessary for symptomatic atrophic vaginitis. | Topical vaginal oestrogen or vaginal oestrogen pessary for symptomatic atrophic vaginitis. | Not changed | - |
|  |  | **Section H: Analgesics** | |  |  |
| 31. | H1 | High-potency opioids in moderate-severe pain, where paracetamol, NSAIDs or low-potency opioids are not appropriate to the pain severity or have been ineffective | High-potency opioids in moderate-severe pain, where paracetamol, NSAIDs or low-potency opioids are not appropriate to the pain severity or have been ineffective | Not changed | - |
| 32. | H2 | Laxatives in patients receiving opioids regularly | Laxatives in patients receiving opioids regularly | Not changed | - |
| 33. |  |  | Short-acting opioids for break-through pain if already on long acting pain relievers for breakthrough pain | Newly added | Increase clarity  and avoid duplication |
|  |  | **Section I: Vaccines** | |  |  |
| 34. | I1 | Seasonal trivalent influenza vaccine annually | Seasonal trivalent influenza vaccine annually | Not changed | - |
| 35. | I2 | Pneumococcal vaccine at least once after age 65 according to national guidelines | Pneumococcal vaccine at least once after age 65 according to national guidelines | Not changed | - |
